# Immune Checkpoint Blockade Response in Mucinous Tubular and Spindle Cell Carcinoma

**DOI:** 10.3390/curroncol32020094

**Published:** 2025-02-08

**Authors:** Simran Makker, Neil J. Shah, Maria I. Carlo, Fengshen Kuo, A. Ari Hakimi, Ying-Bei Chen, Gopa Iyer, Ritesh R. Kotecha

**Affiliations:** 1 Laurel Springs School, Ojai, CA 93023, USA; 2 Department of Medicine, Memorial Sloan Kettering Cancer Center, New York, NY 10065, USAiyerg@mskcc.org (G.I.); 3 Immunogenomics & Precision Oncology Platform, Memorial Sloan Kettering Cancer Center, New York, NY 10065, USA; 4 Department of Surgery, Memorial Sloan Kettering Cancer Center, New York, NY 10065, USA; 5 Department of Pathology and Laboratory Medicine, Memorial Sloan Kettering Cancer Center, New York, NY 10065, USA

**Keywords:** MTSCC, mucinous tubular and spindle cell carcinoma, PD-1

## Abstract

Mucinous tubular and spindle cell carcinoma (MTSCC) is a rare kidney tumor which is usually characterized by indolent disease physiology. While several high-grade and sarcomatoid MTSCC tumors have been reported, the clinical experience with contemporary immune checkpoint blockade (ICB) combination therapies extrapolated from treatment paradigms of conventional renal cell carcinoma (RCC) remains limited. Here, we report two patients with metastatic MTSCC treated with first-line ipilimumab plus nivolumab therapy who both achieved great clinical benefit. We subsequently performed immune deconvolution analysis on previously identified MTSCC-like kidney tumors from The Cancer Genome Atlas (TCGA) and discovered significantly higher PD-L1 transcriptomic expression compared to similar papillary RCC tumors, providing additional biomarker data supporting the observed ICB response. These data implicate ICB therapy as an effective treatment for patients with metastatic MTSCC.

## 1. Introduction

Renal cell carcinomas (RCCs) comprise a heterogenous group of malignancies with increasingly defined clinical, pathological and molecular features. Mucinous tubular and spindle cell carcinoma (MTSCC), a renal tumor subtype first introduced in the 2004 World Health Organization (WHO) classification of renal tumors, is a rare tumor variant with overlapping features with papillary renal cell carcinoma (pRCC). While this disease entity is usually regarded as an indolent tumor with a favorable prognosis [1,2,3,4,5], high-grade and sarcomatoid variant MTSCC tumors have been described with variable responses to systemic therapy. For instance, in a large retrospective review of 25 MTSCC patients treated over more than 10 years, only six patients (24%) received systemic therapy for metastatic disease, of which only three patients received anti-PD-1 therapy in the third-line setting [2].

With the advancement of combination immune checkpoint blockade (ICB) strategies to the first-line setting for clear cell RCC (ccRCC), including ipilimumab plus nivolumab or combination ICB plus VEGF tyrosine kinase inhibitor (TKI) therapy, treatment of rare non-clear cell RCC variants has been extrapolated from these data despite differing tumor biology. One previous case report described a patient with metastatic MTSCC to bone treated with first-line ipilimumab plus nivolumab with a complete response [6], and other reports detail an anti-PD-1 monotherapy benefit in the refractory setting after TKI therapy. Here, we describe two patients with metastatic MTSCC treated with first-line combination ipilimumab plus nivolumab who experienced clinical benefit. We discuss these clinical experiences with pathology and available somatic molecular data. As prior efforts have shown that some pRCC tumors (KIRP) within The Cancer Genome Atlas (TCGA) harbor molecular similarities to MTSCC [7], we performed immune deconvolution analysis to compare hallmark gene enrichment of these MTSCC-like tumors to other KIRP tumors to identify robust biomarkers supportive of response to ICB therapy.

## 2. Case Presentations

### 2.1. Case 1

**Figure 1:**
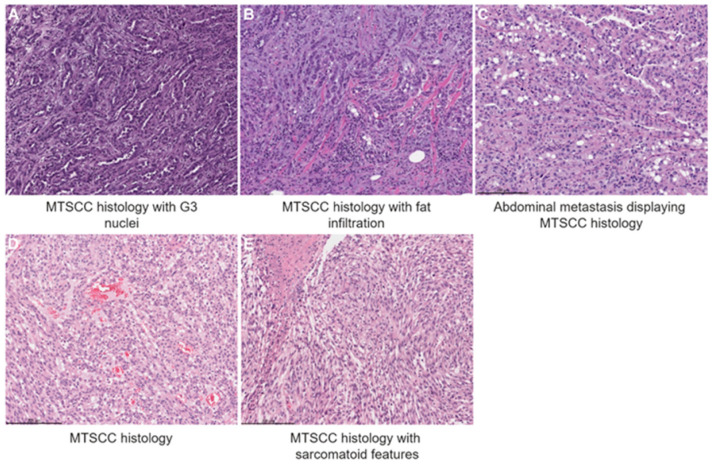
Microscopic examination of MTSCC with high-grade histologic features. Case 1 (**A**–**C**) shows high nuclear grade, poorly differentiated tubules and individual tumor cells infiltrating renal sinus fat (hematoxylin and eosin stain) and Case 2 (**D**,**E**) shows sarcomatoid features. Scale bar = 200 μm.

A 68-year-old woman with a long-standing history of a right renal cyst presented with a growing scalp lesion. Baseline hemoglobin was 14.9 g/dL, serum creatinine 0.6 mg/dL (EGFR > 60 mL/min) and other remaining lab parameters were within normal limits. A biopsy of this lesion showed carcinoma with glandular/ductal features in the dermis. A contrast-enhanced CT of the chest, abdomen and pelvis showed a 6.6 × 5.7 × 5.4 cm right upper pole cystic renal mass with thickened internal septations and mural nodularity. An additional left-sided renal lesion was found, which subsequently was confirmed as an unrelated angiomyolipoma, and there was also an indeterminate small subpleural sub-centimeter nodule in the right lung base. She then underwent a right radical nephrectomy which revealed a poorly differentiated carcinoma with grade 4 focal rhabdoid features extending into the perinephric tissues and renal sinus fat (pT3) (Figure 1A–C). Tumor cells were positive for PAX8 and AMACR, while negative for CK7, 34BE12 and SF-1, and have retained SMARCB1 (INI-1), FH, SDHB and BRG-1 expression. SNP-array showed that the tumor harbors chromosomal losses or copy neutral loss of heterozygosity (LOH) involving chromosomes 1, 3, 4, 6, 9, 10, 11, 13, 14q, 15, 16, 21 and 22, consistent with the characteristic genome-wide copy number alterations of MTSCC, supporting a diagnosis of MTSCC with high-grade features. MSK-IMPACT (Memorial Sloan Kettering Cancer Center-Mutation Profiling of Actionable Cancer Target) [8] germline testing was performed and negative, and the somatic panel was noted to be microsatellite instability-intermediate and positive for an *MLH1* intragenic deletion of exons 2-8 and mutations in *ALOX12B*, *ARID1B*, *ASXL1*, *DCUN1D1*, *DROSHA*, *EP300*, *KEAP1*, *KMT2B*, *KMT2D* and *NPM1*. Immunohistochemistry (IHC) confirmed the loss of MLH1 and PMS2 expression. Similarly to SNP-array, FACETS analysis showed copy number losses of chromosome arms 1p, 3, 4, 6, 9, 10, 11,13q, 15q, 18q, 22q, loss of heterozygosity (LOH) of chromosome arms 1q21-42, 21q and chromosome 16, and copy number gains of chromosome arms 1q43-44, 18p and chromosome 20. Notably, laboratory values post-nephrectomy also remained within normal limits including complete blood count and comprehensive metabolic panel.

One month following radical nephrectomy, the patient underwent radical resection of the right femoral head and intertrochanteric region with cryoablation, and pathology was compatible with known MTSCC (PAX8/AMACR+). The patient received palliative radiation therapy (RT) to the right femur and was maintained on active surveillance. Eleven months later, she developed metastases to the right posterior ilium, a retrocaval lymph node and peritoneal carcinomatosis. She was classified as intermediate risk per the International Metastatic RCC Database Consortium (IMDC) risk classification. She received radiation to the ilium and subsequently started systemic therapy with ipilimumab (1 mg/kg) and nivolumab (3 mg/kg). During treatment, she developed immune-mediated hypothyroidism which normalized with levothyroxine supplementation. Therapy was discontinued for grade 4 immune-mediated hepatitis, treated with IV methylprednisolone (2 mg/kg) followed by an 8-week prednisone taper. Imaging 2 months later showed tumor reduction in multiple sites, including peritoneal implant (1.8 × 1.7 cm, previously 2.4 × 2.2 cm), right nephrectomy bed (0.7 × 0.7 cm, previously 1.1 × 0.9 cm) and improved nodularity in the posterior right thyroid. Subsequent imaging, 5 months post-systemic therapy, showed a continued decrease in peritoneal implant (0.8 × 0.7 cm) while off systemic therapy. At 11 months, the patient had continued stability at all disease sites, and at 14 months post-therapy, the patient had minimal asymptomatic progression in a retroperitoneal mass. She underwent resection 19 months post initial ipilimumab plus nivolumab, and pathology revealed poorly differentiated carcinoma. The patient continued active surveillance, and 32 months post ICB therapy, the patient developed a supraumbilical metastasis which was resected and consistent with recurrent MTSCC. Surveillance was continued and 56 months post systemic therapy, the patient developed recurrent MTSCC in a liver mass status post IR ablation. The patient remains well on active surveillance off systemic treatment.

### 2.2. Case 2

A 76-year-old woman initially presented with a kidney mass and underwent a right radical nephrectomy. Laboratory values at that time showed normal baseline complete blood count and comprehensive panel. Pathology showed multifocal, grade 4 MTSCC with sarcomatoid features and extensive hemorrhage, necrosis and histiocytic response, measuring 17 cm (pT2bNXM0). The tumor was diffusely positive for CAM5.2, AE1/AE3, focally positive for PAX8, and negative for CK7, CD10, MART, TTF-1, Desmin, Calretinin, GATA3, ER, Inhibin, CK20 and EMA, with Ki-67 proliferative index > 80% (Figure 1D,E). MMR staining was not performed. The patient was surveilled, and 6 months following nephrectomy, an FDG-PET/CT scan and contrast-enhanced brain MRI revealed a hepatic pericapsular lesion, and an enhancing mass involving the right parietal and left frontal bones with extension to the scalp and extra-axial spaces. The patient was classified as intermediate risk per the IMDC. The patient completed radiation therapy to the brain and initiated ipilimumab (1mg/kg) plus nivolumab (3 mg/kg) induction for four cycles. After initiation of the treatment, the patient was found to have a treatment response with resolution of the perihepatic lesion, resolved left front soft tissue masses and scalp mass, without new areas of disease. The patient continued with nivolumab monotherapy. After seven months, due to progression in a scalp metastasis, the patient was enrolled on a clinical trial of combination VEGFR TKI plus anti-PD-1 plus anti-LAG-3 therapy. Treatment was complicated by immune-mediated hypothyroidism treated with supplemental levothyroxine, and treatment was later discontinued due to a grade 3 rash subsequently managed with topical agents. Given other sites of disease (e.g., brain) which exhibited a continued response, the patient remained on active surveillance for an additional seven months at which point she was found to have worsening chest wall cutaneous lesions. The patient was initiated on lenvatinib plus pembrolizumab with a follow-up CT scan showing progression of the disease, at which time best supportive care was recommended.

## 3. Discussion

We describe two patients with high-grade, metastatic MTSCC treated with first-line ipilimumab plus nivolumab who sustained clinical benefit. The first patient underwent serial metastatectomy for oligo-progressive disease with a continued treatment-free interval; in contrast, the second patient, who also developed oligo-progressive disease after combination ICB therapy was switched to second-line combination treatment with VEGFR TKI and ICB on a clinical trial that was subsequently discontinued due to treatment-related toxicities. The exhibited prolonged clinical benefit, though, was thought to be conferred by initial first-line ipilimumab plus nivolumab therapy. These cases support prior reports demonstrating significant clinical benefit in MTSCC tumors treated with combination dual ICB therapy and support consideration and prospective study of this treatment regimen in this extremely rare RCC tumor subtype.

Several prospective and retrospective reports have highlighted the variability of the efficacy of ICB-based therapy in non-clear cell RCC tumors. Combination ICB/VEGF therapy is associated with the highest overall response rates, with treatment efficacy observed with regimens including cabozantinib plus nivolumab [9] and lenvatinib plus pembrolizumab [10]. Notably, both phase II trials enrolled patients with non-clear cell RCC histologies including papillary, unclassified, translocation-associated and chromophobe subtypes but did not include MTSCC histology. In the CheckMate-920 study, other non-clear cell RCC histologies also were allowed like collecting duct and renal medullary carcinoma but did not include MTSCC patients in their cohorts and thus the overall experience with dual ICB/ICB therapy for non-clear cell histologies has been limited [11,12]. Recent results from SUNNIFORECAST, the first prospective randomized trial of ipilimumab + nivolumab in non-clear cell RCC has gained significant attention [13]. This trial included 178 non-clear cell RCC patients, the majority of whom had papillary RCC (57.6%), and chromophobe RCC (19.4%). Across the whole population, patients with tumors harboring PD-L1 > 1% had a favorable response to the ipilimumab + nivolumab (HR 0.56, 95% CI: 0.33–0.95) preliminary suggesting this as a biomarker in non-clear cell RCC histologies. While the trial included 50 patients with other rare non-clear cell RCC subtypes, it is not known at this time whether any patients had MTSCC [13].

Use of either VEGFR TKI or ICB therapy for MTSCC has shown mixed outcomes in case reports and case series. In a retrospective review of MTSCC patients, variable benefit was seen with VEGFR TKIs including sunitinib, pazopanib, cabozantinib and other VEGF inhibitors such as bevacizumab. Most of these reports, though, include patients with MTSCC and high-grade/sarcomatoid disease and this again may be related to the inherent aggressive nature of such cases. For patients treated with ICB therapy, experience has additionally been variable. In a series of 41 patients with non-clear cell RCC treated with nivolumab, one patient with metastatic MTSCC had disease progression [14]. In another report, a patient who experienced progression on VEGFR TKI and subsequently developed a complete response to single agent nivolumab [15]. Fuchizawa et al. reported a case of metastatic PD-L1+, MSS, TMB-low MTSCC with an *FBXW7* nonsense mutation with metastases to the bone treated with ipilimumab plus nivolumab as first-line therapy. After four cycles of combination ICB therapy, the patient received nine cycles of nivolumab followed by delayed cytoreductive radical nephrectomy, with resumption of nivolumab without progressive disease at 15 months, at which point therapy was terminated given no evidence of disease [6]. Chahoud et al. reported on one patient with MTSCC who had stable disease and progression-free survival of 7.4 months on nivolumab [16].

In the context of biomarkers associated with clinical benefit to ICB therapy, several unique features were apparent in our two patients. In the first case, the patient was found to have mismatch repair (MMR) deficiency in the original tumor by molecular profiling. While this patient was included in a larger series of MTSCC patients reported from our institution [2], we present here treatment response data which were not reported previously. MMR deficiency or microsatellite instability (MSI) is not a feature commonly found in RCC tumors but alterations in DNA damage response (DDR) pathways have previously been shown to be associated with ICB response in ccRCC [17]. Our report also included MTSCC tumor with sarcomatoid and rhabdoid features, which often in ccRCC display a highly inflamed subtype characterized by immune activation and PD-L1 expression [18]. It is unknown though whether rhabdoid/sarcomatoid MTSCC tumors display similar molecular findings. Nevertheless, exploratory analyses from CheckMate-214, the phase III registration study of ipilimumab plus nivolumab, showed that ipilimumab plus nivolumab had high overall response and complete response rates in ccRCC with sarcomatoid features [19].

MTSCC tumors are characterized by recurrent chromosomal changes [7]. In contrast to pRCC patients within TCGA and other studies, MTSCC tumors show multiple chromosomal gains including nearly universal gains of chromosomes 7 and 17 and less frequent gains of chromosomes 2, 3, 12, 16 and 20 [20,21]. These findings corroborate results by Mehra et al. [7] and support the notion that MTSCC harbor distinct and characteristic chromosomal copy number alterations. Other groups have shown losses of chromosomes 1, 2, 4 through 10, 12 through 15, 17, 20, 22 and X in MTSCC while lacking in trisomy 7 or 17 [1,4,5,7,21,22,23]. Ged et al. identified a loss of heterozygosity (LOH) of chromosomes 1, 15 and 22 in 100% of cases, 6, 9 and 13 in 80% of cases and 14 in 60% of cases [2]. Given the overall challenge in diagnosing MTSCC tumor types, molecular analysis particularly beyond platform testing to identify copy number alterations may be helpful in discerning profiles associated with MTSCC.

**Figure 2:**
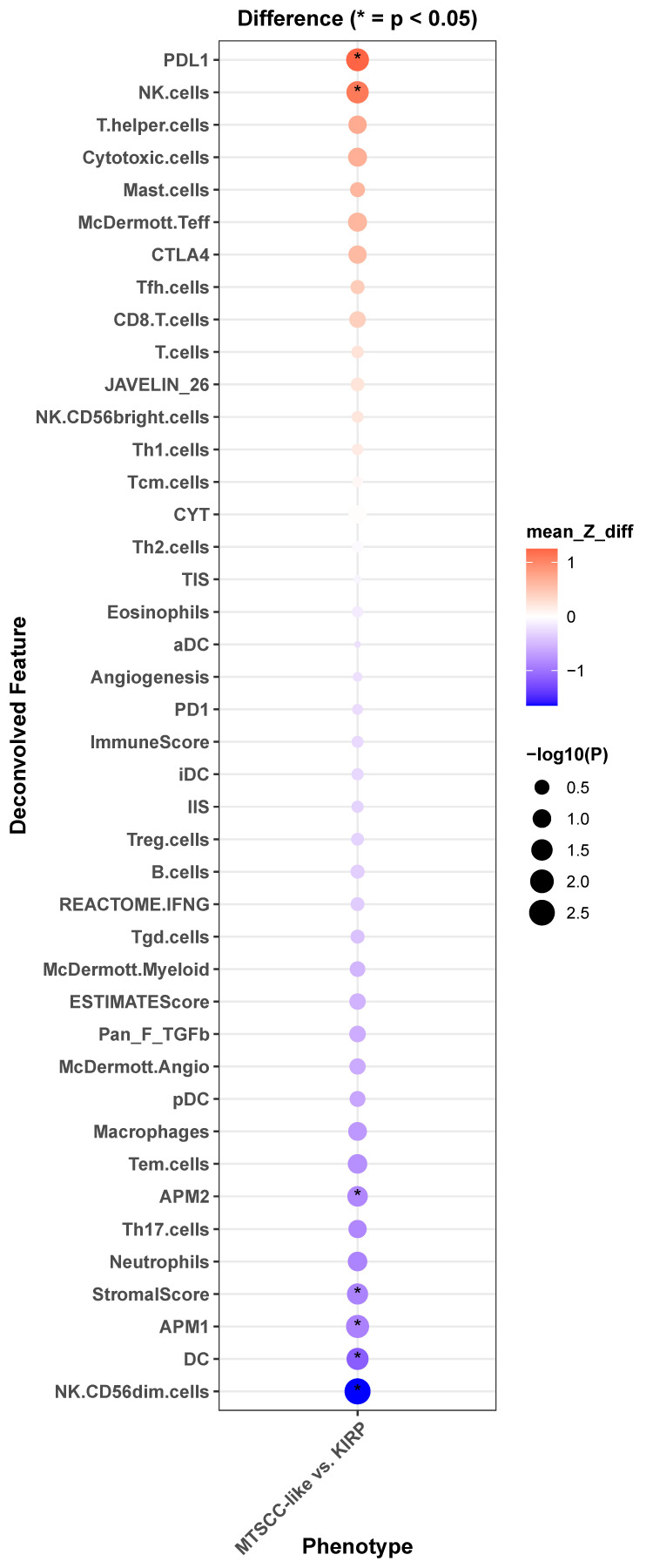
Transcriptomic differences between MTSCC-like and KIRP patients. Gene set enrichment analysis of hallmark gene sets comparing MTSCC-like tumors previously identified from Mehra et al. [7] with other papillary RCC tumors from TCGA KIRP cohort using the log2mean method. Red represents overexpression and blue represents decreased expression. MTSCC-like tumors had significantly higher PD-L1 expression and lower antigen processing machinery signatures (APM1 and AMP2).

An analysis of the pRCC cohort within the TCGA (KIRP) demonstrated that five tumors molecularly resemble an MTSCC phenotype rather than KIRP [7]. We performed immune deconvolution analysis of these MTSCC-like tumors compared to KIRP and discovered significant upregulation of PD-L1 and decreased expression of antigen presentation machinery (APM1, APM2) (Figure 2). This finding is concordant with the prior case report demonstrating high PD-L1 expression by IHC [6]. Interestingly, PD-L1 expression has been shown to be higher in the spindle components relative to tubular components in MTSCC. Also, the expression of PD-L1 is reportedly higher at the surface of sarcomatoid RCC cells compared to non-sarcomatoid RCC cells, regardless of the parent histology or non-sarcomatoid RCC tumor grade [24,25,26]. These findings, in conjunction with the published clinical data, support the observed activity of ICB therapy in PD-L1+ high-grade MTSCC tumors.

Prior efforts profiling MTSCC tumors have shown that almost uniformly all MTSCC patients harbor dysregulation of the Hippo signaling pathway, including alterations in *NF2*, a feature also found in a subset of highly aggressive unclassified RCC tumors [27], as well as alterations in other Hippo-related genes. Prior IHC analysis has shown nuclear YAP1 (90%) and increased YAP protein levels (68%) in MTSCC tumors [7]. These data suggest that Hippo pathway dysregulation may be a seminal event in the pathogenesis of MTSCC and may have diagnostic and therapeutic implications for this malignancy. Targeting the Hippo pathway and YAP/TAZ has been an active interest in conventional RCC tumors given implications of hypoxia inducible factor pathway [28], and these data additionally suggest that particularly non-clear cell RCC like MTSCC may similarly be sensitive to oral Tead1 inhibitors [29], which in turn could be combined with PD-1/PD-L1 agents.

## 4. Conclusions

We report two patients with high-grade metastatic MTSCC who derived clinical benefit from first-line dual ICB therapy. We additionally interrogated public databases to identify biomarkers which may be associated with ICB therapy response and identified that MTSCC-like tumors harbor high PD-L1 expression which may be supportive of ICB therapy in this rare tumor subtype. As the treatment for rare non-clear cell RCC tumor variants is often extrapolated based upon conventional RCC regimen paradigms, increasing our experience and understanding of unique aspects of tumor biology will be critical to tailoring treatment. These cases highlight the potential benefit of contemporary combined ICB therapy in the frontline setting and provide further context for the therapeutic opportunities in this rare tumor subtype which warrant prospective clinical trial study.

## Data Availability

Data are contained within the article.
